# Primary antiphospholipid syndrome presenting as antiphospholipid syndrome nephropathy: a case report

**DOI:** 10.1186/1752-1947-9-28

**Published:** 2015-01-29

**Authors:** Rajitha Asanga Abeysekera, Abdul Wahid Mohomad Wazil, Nishantha Nanayakkara, Neelakanthi VI Ratnatunga, Kaushal Maithree Fernando, Jalitha Thinnarachchi

**Affiliations:** Nephrology and Transplantation Unit, Teaching Hospital, No. 532/6 Siebel Place, Kandy, 20000 Sri Lanka

**Keywords:** Antiphospholipid syndrome, Antiphospholipid syndrome nephropathy, Vasculitis

## Abstract

**Introduction:**

Primary antiphospholipid syndrome can be a difficult diagnosis in the absence of typical clinical features. We describe an unusual presentation of primary antiphospholipid syndrome mimicking vasculitis for which the only diagnostic clue on initial presentation was antiphospholipid syndrome nephropathy.

**Case presentation:**

A 29-year-old Sri Lankan woman presented with features mimicking vasculitis with no obvious clinical features of antiphospholipid syndrome. Classical symptoms of antiphospholipid syndrome only appeared months later. A retrospective analysis showed that the only evidence of antiphospholipid syndrome at her first presentation was antiphospholipid syndrome nephropathy on her renal biopsy.

**Conclusions:**

A high degree of suspicion of antiphospholipid syndrome is needed when patients present with non-specific vasculitis features. It has a broad clinical impact as antiphospholipid syndrome can present to any clinician with rare manifestations such as nephropathy. This significantly adds to the advancement of knowledge as antiphospholipid syndrome nephropathy should be recognized as a true entity and considered as a classification criteria for antiphospholipid syndrome.

## Introduction

Antiphospholipid syndrome (APS) is an autoimmune thrombophilic condition occurring due to the presence of antibodies that recognize phospholipid-binding proteins [1]. APS can be primary or secondary. Primary APS occurs in the absence of any other related disease. Secondary APS occurs with other autoimmune diseases, such as systemic lupus erythematosus (SLE) [1]. Primary APS can be a difficult diagnosis in the absence of typical clinical features. The presentation can vary, mimicking many other medical conditions [2–4]. We describe the case of a patient with primary APS whose initial presentation mimicked vasculitis, the only clue to the correct diagnosis at initial presentation being antiphospholipid syndrome nephropathy (APSN).

## Case presentation

**Figure 1:**
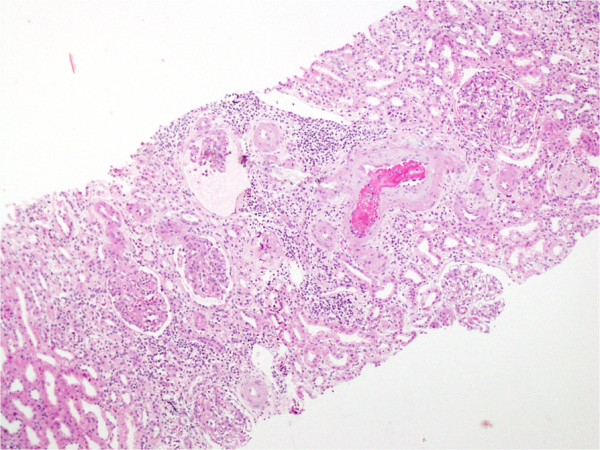
**Renal biopsy, light microscopy, hematoxylin and eosin.** An interlobular artery shows a fresh thrombus. Three glomeruli show ischaemic collapse. The interstitium shows a lymphocytic infiltrate. Afferent arteriole showed fresh thrombi. Magnification 10×10.

A 29-year-old Sri Lankan woman who was previously of good health presented with newly diagnosed hypertension and several recent onset lower limb ulcers which were concluded to be possible vasculitic ulcers following a dermatological review. She had no other symptoms or features of any connective tissue disorder, such as SLE, scleroderma, dermatomyositis or polymyositis. Her examination revealed her blood pressure to be 210/140mmHg. Initial investigations revealed her hemoglobin level to be 9g/dL, with a normal platelet count. Her erythrocyte sedimentation rate was 78mm/hr. Her activated partial thromboplastin time (APTT), immune screen, including anti-nuclear antibodies and anti-neutrophil cytoplasmic antibodies test results were negative. Her urine full report revealed microscopic hematuria and her serum creatinine level was 132μmol/L (normal range: 53 to 116). Her renal and mesenteric angiogram test results were normal. With a probable diagnosis of small- or medium-vessel vasculitis, a renal biopsy (Figure 1) was performed which revealed mesangial hypercellularity, two arteries with fresh thrombi, which was in keeping with the suspected diagnosis of vasculitis. Her immunofluorescence results were negative for immunoglobulin (IgG) and C3, with IgA and IgM 1+ fine granules seen in her capillaries. Immunosuppressive therapy was initiated, however, she was a poorly compliant patient with poor compliance to treatment and follow-up procedures.

**Figure 2:**
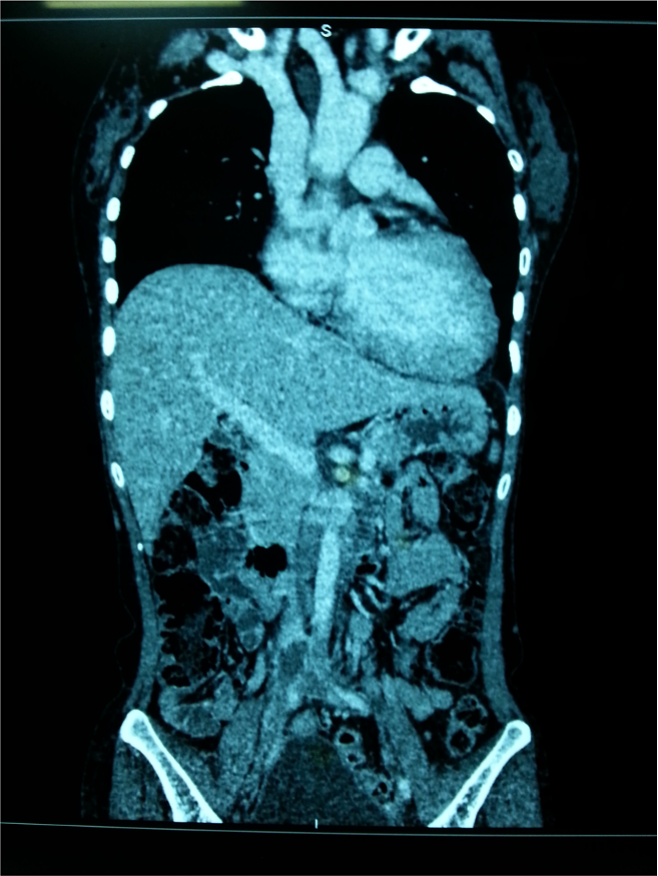
**Computed tomography cavogram.** A 4.8cm segment of thrombosis of the inferior vena cava, extending from just below the renal vein up to the bifurcation.

One year later, she presented with body weakness on her left side, and her computed tomography (CT) brain scan revealed a small acute right parietal intracerebral hemorrhage, which was attributed to her uncontrolled blood pressure. While in the ward she developed an antero-septal ST elevation myocardial infarction, and her coronary angiogram revealed a single 100% occlusion of her left anterior descending artery. Subsequently, over the next few weeks she developed swelling in her right lower limb. These new symptoms prompted a re-evaluation of the previous diagnosis. A duplex scan of her lower limb venous system revealed venous thrombosis in both of her lower limbs, mainly on the right side. A CT cavogram (Figure 2) revealed a 4.8cm segment of thrombosis of her inferior vena cava, extending from just below the renal vein up to the bifurcation. Magnetic resonance imaging of her brain (performed later) revealed multiple small cerebral infarcts with complete resolution of the previous hemorrhage. There were multiple filling defects in her left and right internal carotid arteries, left posterior cerebral artery, left transverse sinus and bilateral sigmoid sinuses, suggestive of thrombi formation. The antiphospholipid antibody (aPL) screening test results were positive for IgG anticardiolipin antibodies with lupus anticoagulant, and a final diagnosis of primary APS was made.

## Discussion

APS is defined by the occurrence of venous or arterial thromboses, or of specific pregnancy morbidity accompanied by the presence of aPL [5]. The clinical presentation of APS at times can be very difficult to diagnose at the first presentation in the absence of classical symptoms. This was the case in our patient, where the initial presentation of APS mimicked vasculitis, and classical symptoms and signs of APS only developed months later. Upon her initial presentation there was no evidence of arterial or venous thrombosis, no pregnancy morbidity and no evidence of hematological involvement such as thrombocytopenia or elevated APTT. A retrospective analysis shows that the only initial clue to the diagnosis were the two thrombi seen in two blood vessels in the renal biopsy, which is a feature of APSN.

The kidney can be a major target organ in APS [6]. Despite this, renal involvement in APS was poorly recognized until recently [7]. Described renal manifestations of APS are renal artery thrombosis and/or stenosis, renal infarction, hypertension, renal vein thrombosis, end-stage renal disease, increased allograft vascular thrombosis and some types of glomerular disease [7]. A small-vessel vaso-occlusive nephropathy was only recently defined as a manifestation of APSN. APSN is characterized by acute thrombotic lesions in glomeruli and/or arterioles (thrombotic microangiopathy) and chronic vascular lesions, such as fibrous intimal hyperplasia of arterioles and interlobular arteries, organized thrombi with or without recanalization and fibrous arterial and arteriolar occlusions or focal cortical atrophy [7, 8]. Studies have shown that APSN patients can have clinical indications such as (often severe), proteinuria (ranging from mild to nephrotic range), hematuria and acute or chronic renal insufficiency [7, 8].

This highlights the presence of these rare symptoms as the presenting feature of APS. The 2006 revised APS classification criteria remarked on some of these clinical features as non-criteria features, which included cardiac valve involvement, livedo reticularis, thrombocytopenia, APSN and non-thrombotic central nervous system manifestations [5]. Some investigators have suggested including APSN in the classification criteria, while remarking that hypertension alone can be first or only clinical indication of APSN [8]. Similarly, there are reported cases where cutaneous ulcers have been the first manifestation of APS [9].

Irrespective of the presentation, anticoagulation with warfarin remains the mainstay of treatment, with an international normalized ratio target of 2.1-3 for the first episode of venous thrombosis, and 3.1-4 being suggested in cases of recurrent thrombotic episodes [10].

## Conclusions

This case highlights the importance of having a high degree of suspicion for APS, especially when the presenting features are non-specific. It has a broad clinical impact as APS can present to any clinician with rare manifestations such as nephropathy. This significantly adds to the advancement of knowledge as APSN should be recognized as a true entity and considered as a classification criteria for APS.

## Consent

Written informed consent was obtained from the patient for publication of this case report and accompanying images. A copy of the written consent is available for review by the Editor-in-Chief of this journal.

## Abbreviations

aPL: Antiphospholipid antibodies
APS: Antiphospholipid syndrome
APSN: Antiphospholipid syndrome nephropathy
APTT: Activated partial thromboplastin time
SLE: Systemic lupus erythematosus.

## References

[1] Rand JH: **The antiphospholipid syndrome.** *Hematology Am Soc Hematol Educ Program.* 2007, **1:**136–42. 10.1182/asheducation-2007.1.136 18024621

[2] Ioannidis P, Maiovis P, Balamoutsos G, Karacostas D: **Primary antiphospholipid syndrome mimicking demyelinating disorders.** *J Neuropsychiatry Clin Neurosci* 2014,**26**(2)**:**E54–5. 10.1176/appi.neuropsych.13050109 10.1176/appi.neuropsych.13050109 24763796

[3] Abdullah AS, Yagoub H, Kiernan TJ, Daly C: **Rapidly progressive coronary artery disease as the first manifestation of antiphospholipid syndrome.** *BMJ Case Rep* 2014. doi:10.1136/bcr-2013–203499 10.1136/bcr-2013-203499 PMC3987570 24713713

[4] Stichlberger M, Lederer W, Wiedermann FJ: **All that seems sepsis is not sepsis: however, is this case report really a case of catastrophic antiphospholipid syndrome?** *Indian J Crit Care Med* 2014,**18**(3)**:**187. 10.4103/0972-5229.128716 10.4103/0972-5229.128716 PMC3963209 24701076

[5] Gómez-Puerta JA, Cervera R: **Diagnosis and classification of the antiphospholipid syndrome.** *J Autoimmun.* 2014, **48–49:**20–5. 10.1016/j.jaut.2014.01.006 24461539

[6] Sciascia S, Cuadrado MJ, Khamashta M, Roccatello D: **Renal involvement in antiphospholipid syndrome.** *Nat Rev Nephrol* 2014,**10**(5)**:**279–89. 10.1038/nrneph.2014.38 10.1038/nrneph.2014.38 24642799

[7] Tektonidou MG: **Renal involvement in the antiphospholipid syndrome (APS)-APS nephropathy.** *Clin Rev Allergy Immunol* 2009,**36**(2–3)**:**131–40. 10.1007/s12016-008-8112-z 19048414

[8] Mubarak M, Nasri H: **What nephrolopathologists need to know about antiphospholipid syndrome-associated nephropathy: is it time for formulating a classification for renal morphologic lesions?** *J Nephropathol* 2014,**3**(1)**:**4–8. 10.12860/jnp.2014.02 PMC3956907 24644536

[9] Aghdashi M, Aghdashi M, Rabiepoor M: **Cutaneous necrosis of lower extremity as the first manifestation of catastrophic antiphospholipid syndrome.** *Mod Rheumatol* 2014. doi:10.3109/14397595.2014.882222 10.3109/14397595.2014.882222 24533540

[10] Lim W: **Antiphospholipid syndrome.** *Hematology Am Soc Hematol Educ Program* 2013, **1:**675–80. 10.1182/asheducation-2013.1.675 24319251

